# Application of mirror reconstruction and 3D printing technology in the treatment of Sanders type IV calcaneal fractures

**DOI:** 10.3389/fsurg.2025.1612958

**Published:** 2025-08-28

**Authors:** Xiong Liao, Jianliang Deng, Di You

**Affiliations:** Department of Orthopedics and Traumatology, The Affiliated Changsha Central Hospital, Hengyang Medical School, University of South China, Changsha, Hunan, China

**Keywords:** 3D printing, calcaneus, fracture, mirror reconstruction, fracture fixation

## Abstract

**Objective:**

To explore the clinical efficacy of an integrated workflow combining mirror reconstruction and 3D printing for Sanders type IV calcaneal fractures—a severely comminuted subtype with limited evidence-based solutions and to explore a new individualized and accurate method for the treatment of Sanders type IV calcaneal fractures.

**Methods:**

A retrospective analysis of the clinical data of 20 patients with Sanders type IV calcaneal fractures who were treated with mirror image reconstruction and 3D printing technology in the Department of Orthopedics of our hospital from April 2021 to July 2023 was performed. There were 16 males and 4 females; their ages ranged from 38 to 62 years, with an average of 48.00 ± 1.84 years. All patients underwent clinical and radiological evaluation. The operation time, intraoperative blood loss, fracture healing time, and surgical complications were recorded. The width and height of the calcaneus, Böhler angle, and gissane angle were measured and compared before surgery, after surgery, and at the last follow-up. At the final follow-up, the American Orthopedic Foot and Ankle Society (AOFAS) ankle and hindfoot scores were used to evaluate hindfoot function, and the pain visual analog scale (VAS) was used to evaluate pain.

**Results:**

All 20 patients in this group underwent successful operations. The operation time ranged from 52 to 75 min, with an average of 59.55 ± 1.52 min. The volume of intraoperative blood loss ranged from 35 to 50 ml, with an average of 41.00 ± 1.16 ml. All patients received satisfactory follow-up, with follow-up times ranging from 12 to 38 months and an average of 16.55 ± 1.34 months. All the fractures healed, and the healing time ranged from 10 to 13 weeks, with an average of 11.55 ± 0.211 weeks. Two patients developed symptoms of sural nerve injury after surgery, two patients developed subtalar joint stiffness after surgery, and two patients developed traumatic arthritis changes in the calcaneellar joint during the 1-year follow-up. At the final follow-up, the calcaneal length, width, height, Böhler angle, and Gissane angle were significantly greater than those before surgery (*p* < 0.05). At the last follow-up, the AOFAS score ranged from 70 to 100 points, with an average of 88.15 ± 2.04 points, of which 8 cases were excellent, 10 were good, and 2 were fair, with an excellent and good rating of 90%. The VAS score ranged from 0 to 3 points, with an average of 0.95 ± 0.22 points.

**Conclusion:**

This integrated approach enables precise reduction with superior short-term outcomes, though long-term validation requires RCTs.

## 1 Introduction

Sanders type IV calcaneal fractures represent <10% of calcaneal injuries, which often leads to complete loss of the calcaneal shape and severe soft tissue damage to the foot and ankle. It can also be accompanied by multiple injuries to the spine, pelvis, etc., making treatment very difficult (1). If treated improperly, various complications, such as traumatic subtalar arthritis and subtalar joint stiffness, are likely to occur, which may require subtalar joint fusion surgery (2). Therefore, open reduction and internal fixation are usually required to reconstruct the joint surface and perform hindfoot alignment to obtain good function (3).

However, owing to the severe comminution of Sanders type IV calcaneal fractures, reduction landmarks are often lacking, making it difficult to achieve anatomical reduction. Therefore, treatments need to be more precise and individualized. In recent years, 3D printing technology has been gradually used in the minimally invasive surgical treatment of Sanders type II and III calcaneal fractures (4), but but evidence for Sanders IV remains scarce. We address this gap by integrating: a. Mirror reconstruction: Unaffected side as anatomical template; b. Virtual reduction planning: Simulated fragment reassembly; c. Physical 3D models: Intraoperative guidance and plate precontouring. This study is among the first to systematize this workflow exclusively for Sanders IV fractures.

This study retrospectively analyzed the clinical data of 20 patients with Sanders type IV calcaneal fractures who were treated with mirror image reconstruction and 3D printing technology in the Orthopedics Department of our hospital from April 2021 to July 2023. Through the evaluation of clinical efficacy, this study aimed to explore an individualized, precise, new method for the treatment of Sanders type IV calcaneal fractures.

## 2 Materials and methods

### 2.1 General information

Sample size justification: Sanders IV fractures are rare (<10% (1). Our cohort (*n* = 20) aligns with similar studies [Cianni et al. (5): *n* = 10; Ozturk et al. (2): *n* = 15] and achieved statistical power >80% for primary endpoints (*α*=0.05).

The inclusion criteria for patients were as follows: ① closed Sanders type IV calcaneal fracture, clearly diagnosed by preoperative CT; ② unilateral isolated calcaneal fracture; ③ age ≥ 18 years; ④ application of mirror reconstruction and 3D printing technology to assist in treatment; and ⑤ complete follow-up data and a follow-up time of 12 months or more. The patient exclusion criteria were as follows: ① severe underlying disease; ② previous ipsilateral calcaneal fracture; ③ previous ipsilateral calcaneal surgery; and ④ pathological fracture.

A total of 20 patients were included, including 16 males and 4 females aged 38–62 years, with an average age of 48.00 ± 1.84 years. There were 18 cases of injuries caused by falling from heights and 2 cases of injuries caused by car accidents. There were 11 patients with spinal fractures and 6 patients with pelvic fractures. All patients signed informed consent forms.

### 2.2 Mirror reconstruction and preoperative surgical simulation

**Figure 1:**
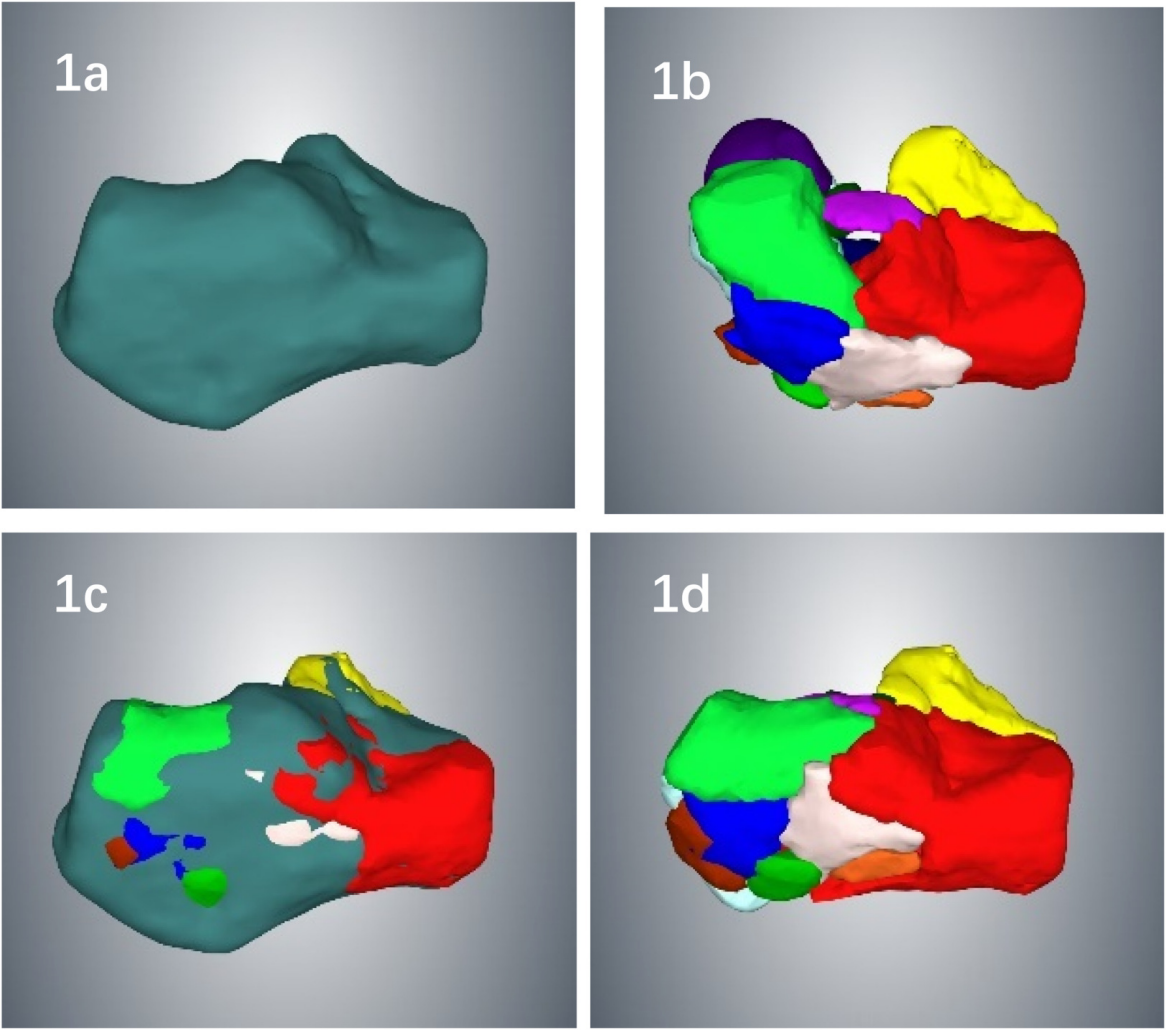
**(a)** using mirror image reconstruction technology, the normal calcaneus model of the affected side is reconstructed using the unaffected calcaneus; **(b)** the calcaneus model of the affected side, with all fracture fragments marked in different colors to facilitate simulated reduction; **(c)** the mirror-image calcaneus model of the unaffected side is used as a template to perform simulated reduction of the fracture; after reduction, an overlapping proportion check is performed to evaluate the quality of the reduction; **(d)** after the reduction is completed, the calcaneal fracture model is close to anatomical reduction.

The raw data of the bilateral calcaneal CT scans (scanning layer thickness of 0.625 mm, we recommend ≤1.5-mm slices to ensure fragment edge clarity.) were exported in DICOM format, and Mimics 20.0 software was used for three-dimensional reconstruction and design. ① Three-dimensional models of the affected and healthy sides of the calcaneus are reconstructed. ② Using mirror reconstruction technology, the normal three-dimensional calcaneal model of the affected side is mirrored through the three-dimensional calcaneal model of the unaffected side (Figure 1a). ③ All the fracture fragments in the three-dimensional model of the calcaneus on the affected side were marked with different colors (Figure 1b). ④ Use the healthy side mirror model as a template to perform simulated reset. After the reduction was completed, the mirror image model of the healthy side and the calcaneus model of the affected side after reduction were used for overlapping matching inspection to evaluate the quality of reduction (Figure 1c). ⑤ The above model is output in the STL format, imported into a 3D printer, and a 1:1 solid model is printed using the nylon polymer as the raw material (Figure 1d). See Figure 1 for details.

### 2.3 3D printing and preoperative plate preshaping

**Figure 2:**
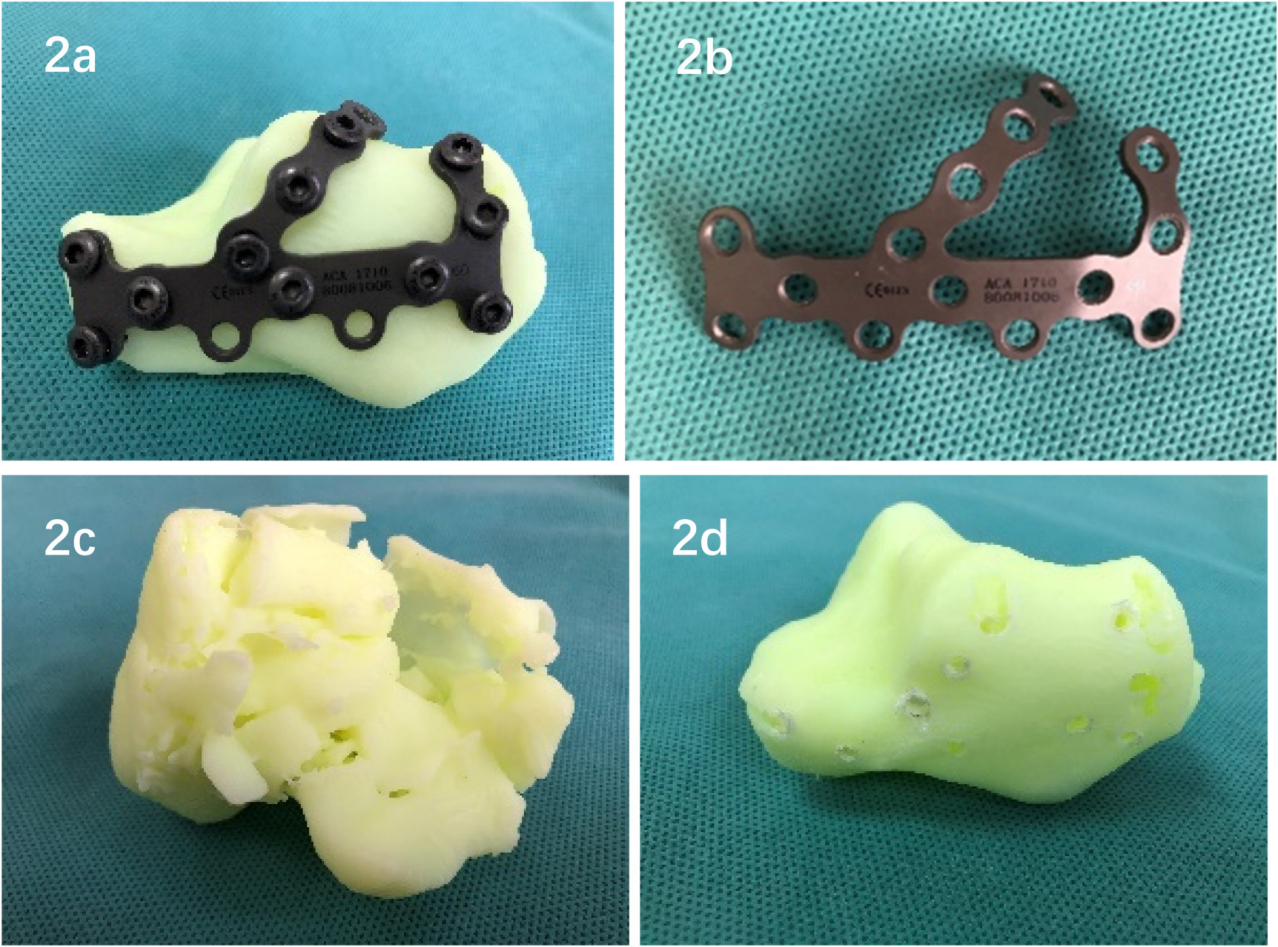
**(a)** perform preoperation on the mirrored solid model of the healthy side of the calcaneus; determine the position of the bone plate; preshape the bone plate; and record the position, length and direction of the screw placement. **(b)** Place the precontoured plateand select screws for direct use during surgery. **(c,d)** The calcaneus fracture model on the affected side and the calcaneus mirror model on the contralateral side are used for real-time observation and guidance during surgery.

Using 3D printing technology, a mirror-image solid model of the unaffected calcaneus is printed, and the model is used as a template to select a bone plate of appropriate size. The bone plate is placed in the ideal position according to the actual fracture shape and presshaped to determine the location of the screws, and the screws are inserted one by one to fix them. The position, length and direction of screw placement were measured and recorded (Figure 2a). The precontoured plate was sterilized, and screws were inserted under high temperature and high pressure for later use. During the operation, the quality of fracture reduction can be judged by the fit between the bone plate and the calcaneus bone after reduction (Figure 2b). The fracture model of the affected side and the calcaneus mirror model of the contralateral side were brought into the operating room for real-time observation and guidance during the operation (Figure 2c, d). See Figure 2 for details.

### 2.4 Surgical methods

All surgeries were performed by the same experienced surgeon. General anesthesia or epidural anesthesia is used, with the patient lying on the healthy side and a tourniquet applied. The classic lateral L-shaped enlarged approach was used. After the incision is made, the lateral wall of the calcaneus is sharply separated, and the skin, subcutaneous and fascia on the lateral side of the foot are completely freed from the calcaneus. Three K-wires are placed on the cuboid bone, talus neck and lateral malleolus in sequence, and the skin flap on the lateral side of the foot is removed. The whole body was retracted to expose the lateral surface of the calcaneus. On the basis of the preoperative surgical planning and intraoperative real-time observation of the 3D printed model, the fracture fragments were sequentially reduced and temporarily fixed with Kirschner wires. The precontoured plate was placed in the preoperatively planned position. The fit between the bone plate and the calcaneus after reduction reflects the quality of fracture reduction well. C-arm fluoroscopy takes lateral, axial and Broden views of the calcaneus to confirm that the reduction is satisfactory and that the bone plate is in good position. The preoperatively selected screws were inserted for fixation according to the preoperatively planned screw placement position and direction, and C-arm fluoroscopy was performed again to confirm that the internal fixator was in a good position. After the incision was flushed and the bleeding was stopped strictly, a wound drainage tube was placed, the fascia was sutured with absorbable sutures, and the skin was sutured with Allogower–Donati sutures. The samples were covered with a dressing, and a moderate-pressure bandage was applied. No cast is used after surgery.

### 2.5 Postoperative management, follow-up and evaluation

Multimodal combined analgesia was given postoperatively. After surgery, patients were encouraged to perform active and passive activities of the ankle and foot and resistance strength training of the lower limbs. Starting from the 8th week after surgery, the affected foot was allowed to undergo progressive partial weight-bearing walking with the assistance of crutches. At 10 to 12 weeks after the operation, on the basis of the fracture healing indicated by x-rays, walking without crutches and full weight-bearing began. The operation time, intraoperative blood loss, fracture healing time, and surgical complications were recorded. The patient was instructed to visit the outpatient clinic for follow-up consultation at 1 month, 2 months, 3 months, 6 months, 12 months, and 24 months after the operation to guide functional exercises and observe the healing of the fracture. Lateral and axial x-rays and three-dimensional CT examinations of the calcaneus were performed before surgery, on the first day after surgery and at the last follow-up. The length, width and height of the calcaneus, Böhler angle, and Gissane angle were used to evaluate the surgical effect. At the last follow-up, hindfoot function was evaluated via the American Orthopedic Orthopedic Surgery Society (AOFAS) ankle and hindfoot score, and pain was assessed via the visual analog scale (VAS).

### 2.6 Statistical processing

Statistical analysis was performed via SPSS 22.0 statistical software. In the present study, age, intraoperative bleeding, operation time, fracture healing time, follow-up time, preoperative and postoperative calcaneal length, calcaneal width, calcaneal height, calcaneal Böhler angle, calcaneal Gissane angle, VAS score and AOFAS score were continuous variables and are expressed as the mean and standard deviation (mean ± SD). Two tests, the Kolmogorov‒Smirnov test and the Shapiro‒Wilk test, were used for normality testing. If *P* > 0.05, the data met the normal distribution assumption; otherwise, the data did not meet the normal distribution assumption. A paired sample Wilcoxon signed rank test (nonnormal distribution) or paired sample t test (normal distribution) was used to analyze the differences in preoperative and postoperative indicators. A *p* value < 0.05 was considered statistically significant.

## 3 Results

**Figure 3:**
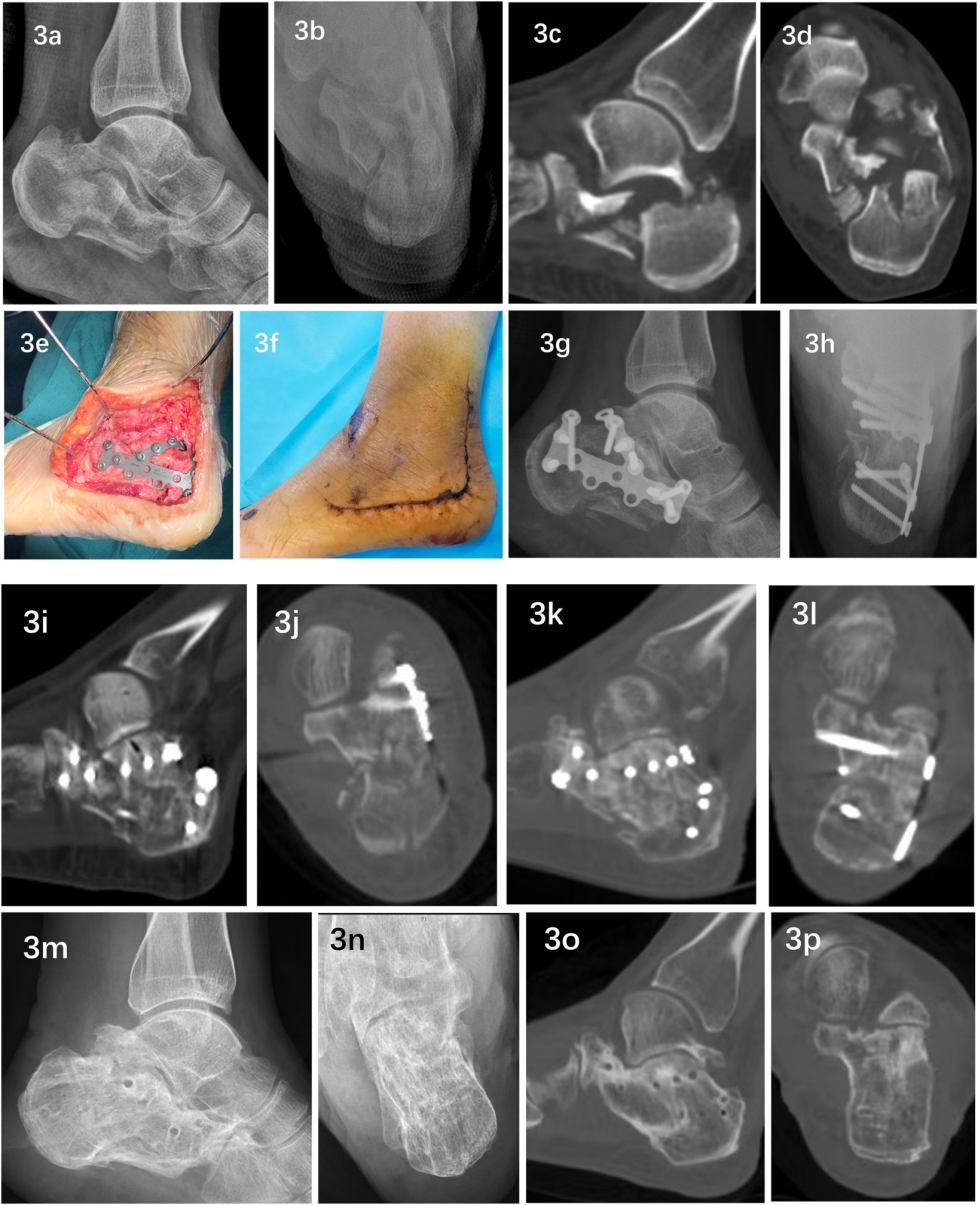
Typical case: A 39-year-old female experienced a closed fracture of the left calcaneus (Sanders type IV) due to a fall from a height combined with a burst fracture of the third lumbar vertebra and no neurological symptoms in either lower limb. Preoperative lateral and axial x-rays **(a,b)** and sagittal and axial CT scans **(c,d)** revealed that the calcaneus was highly comminuted and that its shape was basically lost. After admission, emergency open reduction and internal fixation of the third lumbar vertebral body burst fracture were performed. On the 17th day after admission, with the assistance of mirror reconstruction and 3D printing technology (Figures 1, 2), open reduction and internal fixation through the lateral L-shaped expanded approach were performed. During the operation, a precontoured plate and reduced bone were observed. The calcaneus fits well (e) The operation took 55 min, and 45 ml of blood was lost during the operation. The incision healed in the first stage after the operation **(f)** Postoperative lateral and axial x-rays **(g,h)** and sagittal and axial CT scans **(i,j)** revealed that the length, width, height, Böhler angle and Gissane angle of the calcaneus were completely restored, and the posterior subtalar articular surface was completely restored. Collapse and displacement were completely corrected, the articular surface fracture gap was <1 mm, and the varus deformity was completely corrected. The patient was discharged from the hospital on postoperative day 14 and was followed up for 38 months. Twelve weeks after surgery, sagittal and axial CT scans **(k,l)** revealed complete fracture healing without loss of reduction. Thirty-eight months after the operation, the internal fixator was removed at the patient's request. Lateral and axial x-ray films **(m,n)** and sagittal and axial CT scans **(o,p)** revealed no loss of reduction and no obvious calcaneotalar joints. Signs of traumatic arthritis. The AOFAS score at the last follow-up was 96 points, and the VAS score was 0 points.

All 20 patients in this group completed the operation successfully, with operation times ranging from 52 to 75 min and an average of 59.55 ± 1.52 min. The volume of intraoperative blood loss ranged from 35 to 50 ml, with an average of 41.00 ± 1.16 ml. The surgical incisions all healed at first sight. Postoperative x-rays and CT scans revealed that the fracture end was in a satisfactory position and that the screws were in a good position; all patients received satisfactory follow-up, with a follow-up time ranging from 12 to 38 months, with an average of 16.55 ± 1.34 months. All the fractures healed smoothly, and the healing time ranged from 10 to 13 weeks, with an average of 11.55 ± 0.211 weeks. Two patients developed symptoms of sural nerve injury after surgery, manifesting as local pain and numbness. The symptoms disappeared after local sealing treatment. Two patients developed subtalar joint stiffness after surgery, which manifested as limited active inversion and eversion of the foot. The patients basically recovered after systematic rehabilitation training in the rehabilitation department. Two patients developed traumatic arthritis changes in the calcaneellar joint during the 1-year follow-up. At the final follow-up, the calcaneal length, width, height, Böhler angle, and Gissane angle were significantly greater than those before surgery (*p* < 0.05). See Table 1 for details. At the last follow-up, the patient's hindfoot alignment was normal, with no varus and valgus deformities, no loss of reduction, and no screw loosening or breakage. At the last follow-up, the AOFAS score ranged from 70 to 100 points, with an average of 88.15 ± 2.04 points, of which 8 cases were excellent, 10 were good, and 2 were fair, with an excellent and good rating of 90%. The VAS score ranged from 0 to 3 points, with an average of 0.95 ± 0.22 points. There was no pain or only mild pain. See Table 2 for details. A typical case is shown in Figure 3.

**Table 1:** Comparison of imaging measurement results before surgery and at last follow-up.

Parameter	Preoperative value	Postoperative value	*P*-value
Length of calcaneus（mm）	71.90 ± 0.63	73.60 ± 0.58	<0.01
Width of calcaneus（mm）	37.57 ± 0.31	34.13 ± 0.25	<0.01
Height of calcaneus（mm）	42.13 ± 0.53	47.42 ± 0.49	<0.01
Calcaneal Böhler angle（°）	−0.61 ± 3.15	34.43 ± 1.16	<0.01
Calcaneal Gissane angle（°）	164.86 ± 1.83	134.16 ± 1.61	<0.01

**Table 2:** Functional and pain scores at last follow-up.

Score	Mean ± SD	Excellent（90–100）		Good（75–89）		Average（50–74）	
		Mean ± SD	Number of cases（%）	Mean ± SD	Number of cases（%）	Mean ± SD	Number of cases（%）
AOFAS	88.15 ± 2.04	97.13 ± 2.75	8（40）	84.40 ± 4.30	10（50）	71.00 ± 1.41	2（10）
VAS	0.95 ± 0.22	-					

## 4 Discussion

### 4.1 Current challenges in the treatment of Sanders type IV calcaneal fractures and the problems this study attempts to solve

Sander type IV calcaneal fractures still pose a challenge to orthopedic surgeons because they often lead to poor clinical outcomes and poor quality of life, and there is a lack of consensus on the optimal treatment plan (5). Sander type IV calcaneal fractures are prone to postoperative complications. Complications such as symptomatic subtalar arthritis and arch collapse occur, resulting in poor hindfoot function and eventually leading to subtalar arthrodesis (6). Although subtalar arthrodesis is also an effective option for the treatment of Sanders type IV calcaneal fractures, this technique is recommended when anatomic reduction and fixation are not feasible (7). Most Sanders type IV calcaneal fractures are highly comminuted. Not only are there complex displaced fractures, but the integrity of the articular cartilage is sometimes lost. It is very difficult to achieve anatomic reduction, and the prognosis of surgery is poor (8, 9). Therefore, the difficulties in the surgical treatment of Sanders type IV calcaneal fractures are how to achieve or approach anatomical reduction and how to judge the overall calcaneal reduction quality during surgery. This requires an accurate understanding of fracture characteristics such as the number, location, and displacement pattern of calcaneal fragments before surgery. Although three-dimensional CT reconstructions can yield 3D images of calcaneal fractures, there are many limitations in observing three-dimensional complex fractures on a two-dimensional screen, and 3D printing models can display all the characteristics of fractures more intuitively and vividly (10). It is generally believed that the bones of both limbs of the human body are essentially symmetrical, and when fractures are treated, the corresponding bones on the affected side can be used as a reference (11). This study uses the mirror image model of the unaffected calcaneus as a template to plan precise reduction and evaluate the quality of reduction through simulated surgery via virtual software and performs presurgery on the 3D printed mirror image model of the unaffected calcaneus to determine the internal best position of the fixation and its parameters to solve this problem.

### 4.2 Innovations in the application of mirror reconstruction and 3D printing technology to assist in the treatment of Sanders type IV calcaneal fractures

The innovations of this study are as follows: ① The fracture shape and displacement pattern can be clearly understood. Completing a three-dimensional reconstruction of the fracture and segmenting each fragment is a valuable tool and key to preoperative surgical planning (1, 12). After we complete the three-dimensional reconstruction of the fracture model, we mark all the calcaneal fracture fragments in sequence with different colors. By segmenting each fracture fragment one by one, we can conduct 360-degree observations and accurately reproduce the actual fracture shape and fracture displacement. pattern, which is very helpful for obtaining a preoperative understanding of complex fracture morphology and planning precise reduction; ② complete fracture reduction planning through virtual surgery. We used the mirror image model of the unaffected side of the calcaneus as a template to perform simulated reduction. Finally, the fracture model after reduction was compared with the unaffected side mirror image model for overlapping matching inspection to evaluate the quality of reduction. Good overlap indicates good reduction quality (13); 3. 2462;. Determining the optimal position and parameters of the implant presurgery. Presurgery is performed on the 3D-printed mirror-image solid model of the unaffected calcaneus. A bone plate of appropriate size is placed in the optimal position according to the actual shape of the fracture, which is preshaped, and screws are sequentially inserted for fixation. The position, length and direction of the screw were measured and recorded. During the operation, not only can the quality of fracture reduction be indirectly judged by the fit between the bone plate and the calcaneus after reduction, but firm fixation can also be quickly completed according to the preoperatively planned bone plate and screw positions, screw lengths, and screw placement directions. The efficiency of surgery has also significantly improved.

### 4.3 Analysis of the clinical efficacy of using mirror reconstruction and 3D printing technology to assist in the treatment of Sanders type IV calcaneal fractures

Zheng W (14) et al. compared conventional surgery with 3D printing technology-assisted surgery in the treatment of calcaneal fractures and reported that the operation time of the 3D group was significantly shorter than that of the conventional group and that the degree of intraoperative blood loss was also statistically significant. Park HJ et al. (15) used a lateral L-shaped expanded approach to surgically treat Sanders type IV calcaneal fractures. Compared with that of the conventional group, the operation time of the 3D printing model group was shorter, with an average of 82.3 ± 13.2 min, and it was not easy. A lost reset has occurred. This finding is consistent with our research results. In our study, the average amount of intraoperative blood loss was 41.00 ± 1.16 ml. Our operation time was shorter, with an average of 59.55 ± 1.52 min. Operative time reduction stems from precontoured plates eliminating intraoperative trial-and-error. At the last follow-up, there was no loss to reduction, and no cases of screw loosening or breakage were found during the follow-up. Biomechanical studies have shown that the distance between the bone plate and the bone should be less than 2 mm to maintain maximum mechanical stability (16). We preshape the bone plate before surgery to ensure the fit between the bone plate and the reduced heel fracture during the operation and place the bone plate and screws in the best position according to the actual shape of the fracture to ensure good internal fixation. Initial stability. The abovementioned good results are due to the use of preoperative simulation surgery and preoperation on the 3D solid model, which significantly improved surgical efficiency and reduced intraoperative blood loss accordingly. In this study, 2 patients developed symptoms of sural nerve injury after surgery, and 2 patients developed subtalar joint stiffness after surgery. After symptomatic treatment, both patients basically recovered. In addition, 2 patients developed traumatic arthritis changes in the calcaneellar joint during the 1-year follow-up. The rates of these complications were not greater than those reported in other similar studies.

Studies have shown that the clinical outcome of Sanders type IV fractures is not related to the number of fracture fragments but rather to the degree of loss of the Böhler angle, Gissane angle, and calcaneal height. It is very important to restore these parameters during surgery (8). In this study, the following three aspects were used to assess the quality of fracture reduction: 1. 2460;. Preoperatively, the fracture model after reduction was coincident with the mirror image model of the healthy side of the calcaneus; ② intraoperatively, the precontoured plate and the fit of the calcaneus after reduction were used; and 3. 2462;. intraoperative fluoroscopy was performed. In the study by Schepers T et al. (11), the Böhler angle of the 3D group (31.7 ± 5.0°) was significantly greater than that of the conventional treatment group (27.5 ± 4.3°). In our current study, although there was no comparison with the conventional group, the Böhler angle, Gissane angle, and calcaneal height were significantly restored in all patients compared with the preoperative values (*p* < 0.0001). In addition, the length of the calcaneus was significantly restored, and the width of the calcaneus was also significantly narrowed (*p* < 0.0001). This is due to the use of preoperative planning and intraoperative guidance assisted by mirror reconstruction and 3D printing technology, which can ensure the quality of reduction.

Cianni L et al. (5) conducted a retrospective analysis of 10 cases of Sanders type IV fractures in which conventional open reduction and internal fixation were used and completed 6 years of follow-up. They reported that 60% of patients with AOFAS scores had a poor prognosis, and 40% had a poor prognosis. The prognosis is fair. Our excellent and good rate is 90%, which may be related to the precise reduction and good fixation achieved with the assistance of mirror reconstruction and 3D printing technology. However, this was a short-term follow-up result, and 2 patients had already developed traumatic arthritis changes in the calcaneotalar joint. The short- and medium-term average scores cannot yet be considered favorable outcomes (5), and long-term follow-up studies are needed. The comparison results with relevant literature results are shown in Table 3.

**Table 3:** Comparative outcomes vs. key literature.

Parameter	Our study	Cianni et al. (5)	Park et al. (15)
Operative time（min）	59.55 ± 1.52	-	82.3 ± 13.2
Blood loss (ml)	41.00 ± 1.16	-	80.0
AOFAS excellent/good	90%	60%	-
Reduction loss	0%	25%	15%

### 4.4 Disadvantages of applying mirror reconstruction and 3D printing technology to assist in the treatment of Sanders type IV calcaneal fractures

However, there are drawbacks to this technology. It involves mirror reconstruction of the original data, simulated surgical design and 3D model printing, which takes 3–5 days, has a long learning curve, and increases some costs. However, as far as the treatment of Sanders type IV calcaneal fracture is concerned, once this technology is mastered and used proficiently, it will obviously benefit the patient because it can improve the efficiency of the operation, improve the effect of reduction and fixation, and thus improve the clinical performance. Efficacy.

Our study has several limitations: the sample size is small; as a retrospective analysis, there is a lack of a control group for comparison; the follow-up time is short; our minimum follow-up time is one year; and long-term complications such as subtalar arthritis are difficult to evaluate. Longer follow-up studies are needed; imaging parameters were only compared before and after surgery and were not compared with imaging parameters of the normal calcaneus in healthy feet.

In summary, in the treatment of Sanders type IV calcaneal fractures, mirror reconstruction and 3D printing technology can be used for preoperative planning and intraoperative guidance and can assist in achieving precise reduction and good fixation, with satisfactory clinical results. In addition, future studies will integrate computational biomechanics to simulate load-bearing dynamics and further refine implant positioning.

The integrated mirror-3D approach demonstrates significant advantages for Sanders IV fractures in precision and efficiency. While promising, multi-center RCTs are needed to establish long-term benefit.

## Data Availability

The raw data supporting the conclusions of this article will be made available by the authors, without undue reservation.
